# Mapping the deletion endpoints in individuals with 22q11.2 Deletion Syndrome by droplet digital PCR

**DOI:** 10.1186/s12881-014-0106-5

**Published:** 2014-10-14

**Authors:** Vicki J Hwang, Dianna Maar, John Regan, Kathleen Angkustsiri, Tony J Simon, Flora Tassone

**Affiliations:** Department of Biochemistry and Molecular Medicine, UC Davis, 2700 Stockton Blvd, Suite 2102, Sacramento, CA 95817 USA; Digital Biology Center, Bio-Rad Laboratories, 5731 West Las Positas Blvd, Pleasanton, CA 94588, USA; MIND Institute, UC Davis Medical Center, Wet Lab Room 2418, 2805 50th Street, Sacramento, CA 95817 USA; Department of Pediatrics, UC Davis Medical Center, Sacramento, CA USA; Department of Psychiatry, UC Davis Medical Center, Sacramento, CA USA

**Keywords:** Droplet digital PCR, 22q11DS, qPCR, copy number, LCR

## Abstract

**Background:**

Chromosome 22q11.2 deletion syndrome (22q11DS) is the most common human microdeletion syndrome and is associated with many cognitive, neurological and psychiatric disorders. The majority of individuals have a 3 Mb deletion while others have a nested 1.5 Mb deletion, but rare atypical deletions have also been described. To date, a study using droplet digital PCR (ddPCR) has not been conducted to systematically map the chromosomal breakpoints in individuals with 22q11DS, which would provide important genotypic insight into the various phenotypes observed in this syndrome.

**Methods:**

This study uses ddPCR to assess copy number (CN) changes within the chromosome 22q11 deletion region and allows the mapping of the deletion endpoints. We used eight TaqMan assays interspersed throughout the deleted region of 22q11.2 to characterize the deleted region of chromosome 22 in 80 individuals known to have 22q11DS by FISH. Ten EvaGreen assays were used for finer mapping of the six identified individuals with 22q11DS atypical deletions and covering different regions of chromosome 22.

**Results:**

ddPCR provided non-ambiguous CN measurements across the region, confirmed the presence of the deletion in the individuals screened, and led to the identification of five differently sized and located deletions. The majority of the participants (n = 74) had the large 3 Mb deletions, whereas three had the smaller 1.5 Mb deletions, and the remaining three had an interstitial deletion of different size.

**Conclusions:**

The lower cost, rapid execution and high reliability and specificity provided by ddPCR for CN measurements in the 22q11 region constitutes a significant improvement over the variable CN values generated by other technologies. The ability of the ddPCR approach, to provide a high resolution mapping of deletion endpoints may result in the identification of genes that are haplo-insufficient and play a role in the pathogenesis of 22q11DS. Finally, this methodology can be applied to the characterization of other microdeletions throughout the genome.

## Background

Chromosome 22q11.2 Deletion Syndrome (22q11DS) is the most frequent microdeletion syndrome in humans with an estimated prevalence of 1:3000–1:6000 live births [1-4]. A wide array of phenotypes has been associated with this syndrome including structural malformations (congenital heart disease (CHD), thyroid abnormalities, and hypocalcemia) and neurodevelopmental and neurological deficits (Intellectual Disabilities (ID), Attention Deficit Hyperactivity Disorder (ADHD) and seizures) [5].

The majority of 22q11 deletions arise *de novo* (90%) and only a small minority (10%) is inherited. The high frequency of *de novo* events has been attributed to the high mutation-rearrangement rate associated with several low copy repeat (LCR) regions throughout the 22q deletion region [6,7]. The LCRs found in 22q are larger and have higher sequence similarity than most other LCR regions in the genome, possibly explaining the higher prevalence of 22q11DS in the general population compared to other chromosomal deletion syndromes [8,9]. It is thought that the majority of individuals with 22q11DS have deletion breakpoints located within the 22q11 LCRs. Indeed, 70-80% of these individuals have the 3 Mb deletion (between LCR A and D), 10-15% have a nested 1.5 Mb deletion (between LCR A and B), and the remaining have atypical deletions [7,10-33]. These deletions are likely the results of unequal crossing-over during meiosis involving smaller LCRs within the deleted regions.

Multiple studies have attempted to delineate the deletion endpoints in individuals with 22q11DS; however, the difficulty in designing unique primers or identifying unique SNPs in the proximity or within these repeat regions has hindered the progress of locating the exact position of the deletion endpoints which remains poorly defined [10,34-36].

Several studies have also attempted to elucidate genotype-phenotype correlations among individuals with 22q11DS or to identify a minimal region of disease, but these correlations are still not well understood [12,15]. Reports of individuals with different size deletions that cover nearby, non-overlapping regions and presenting with similar clinical phenotypes stress the importance of delineating the exact deletion endpoints in order to accurately bin individuals and specific phenotypes. Specifically, Amati et al. [14] and Yamagishi et al. [20] described several cases with different, adjacent deletions located between LCR-A and LCR-B that presented with CHD and facial dysmorphism. These observations suggest that a minimal critical region for these phenotypes that exists between LCR-A and LCR-B. The need for better genotype and phenotype correlations in individuals with 22q11DS makes it important to screen a large cohort with high resolution in order to determine the exact deletion breakpoints and to correctly identify those regions that may correlate to specific phenotypes.

So far, the methodologies utilized for gene copy number quantification have often given inconsistent results in target populations. Currently, the gold standard method of diagnosis for 22q11DS is by fluorescent *in-situ* hybridization (FISH) [5,37]. However, this approach is labor intensive, expensive, requires specialized and well trained technicians and equipment, and may not identify a small portion of individuals with 22q11DS. In addition, it is difficult to adopt FISH in a high throughput setting. Other methodologies currently used to determine the presence of a deletion and to delineate the endpoints for 22q11DS include: multiplex ligation-dependent probe amplification (MLPA) [38], quantitative PCR (qPCR) [35], and multiple types of chromosomal arrays (single nucleotide polymorphisms microarrays, (SNPs) and array-based comparative genomic hybridization, aCGH) [39]. However, none of these have been shown to be high-throughput, robust and cost-effective. Specifically, the high cost and technical complexity of the CGH array make it unsuitable for routine diagnostic use and for high throughput testing. Various steps have been found to be critical and limiting for the performance of MLPA including the choice of the reference DNA, incomplete denaturation, and sensitivity to DNA and salt concentration [40,41]. Lastly, qPCR is prone to false positive results, perhaps due to the limited efficiency of the oligonucleotide probes, nucleotide content of the target region and susceptibility to inhibitors [42,43].

Droplet digital PCR (ddPCR) is a new approach to nucleic acid detection and quantification and gives extremely robust values as samples are partitioned into approximately 20,000 water-in-oil droplets, allowing many thousands of discrete measurements to be made. Additionally, as the partitioning of a DNA sample allows for an independent reaction in each droplet, robust CN measurements with 95% confidence can be generated. The binary nature of ddPCR makes it tolerant to differences in PCR amplification efficiency, often allowing for no overlap of 95% confidence intervals for adjacent copy number states [44] and has been demonstrated to possess a high level of accuracy and precision for determining copy number quantification [45]. Finally, as multiplexing can be easily performed using ddPCR, the ~4$ cost per sample allows not only the detection of 22q11DS but simultaneously allows the differentiation between the two common deletions (3 Mb and 1.5 Mb).

In this study, we utilized ddPCR for CN value measurements at different locations within the 22q11.2 region to finely map the deletion breakpoints in 80 individuals with 22q11DS. We demonstrate that ddPCR is a precise and reliable approach for detecting CN variation within the 22q11 locus (using either Taqman or EvaGreen assays) and that ddPCR can be used to delineate the deletion endpoints, which may be helpful in identifying the critical regions corresponding to disease. We also used ddPCR to study genotype-phenotype correlations in 22q11DS and to examine its application for diagnostic and clinical purposes.

## Methods

### Human participants

Participants were recruited at the UC Davis Medical Investigation of Neurodevelopmental Disorders (MIND) Institute located in Sacramento, under written consent from the next of kin, caretakers, or guardians on the behalf of the minors/children participants and according to a UC Davis Institutional Review Board (IRB) approved protocol. The diagnosis of 22q11DS was obtained for the participants by FISH analysis using the *TUPLE1* probe. No information on the size and the position of the deleted region was available. A retrospective blinded study using ddPCR was conducted in order to determine the deletion status of participants with 22q11DS. 95 participants, including 80 individuals (48 males and 32 females) with 22q11DS and 15 TD controls (8 males and 7 females) were chosen. Age range was 7–15 for 22q11DS and 8–14 for TD. Demographic and clinical diagnoses are shown in Table 1.

**Table 1:** **Demographic information and clinical diagnoses**

**Dx**	**No subjects**	**Gender**	**Age range**	**Deletion**		**Severity**	**Phenotypes**					
				**Range**	**Type**		**IQ** **n = 78**	**ADHD** **n = 79**	**Seizures** **n = 79**	**CHD** **n = 80**	**Hypocalcemia** **n = 78**	**Thyroid abnormalities** ** n = 79**
**22q**	80	M = 48 F = 32	M = 7-15 F = 8-15	PRODH-D22S936	3 Mb deletion	Normal	45 (58%)	47 (59%)	59 (75%)	38 (47%)	50 (64%)	62 (78%)
						Abnormal	27 (35%)	26 (33%)	14 (18%)	36 (45%)	22 (28%)	11 (14%)
				PRODH-DGCR6L	1.5 Mb deletion	Normal	1 (1.3%)	0	2 (2.5%)	2 (2.5%)	3 (4%)	3 (4%)
						Abnormal	2 (3%)	3 (4%)	1 (1.3%)	1 (1.3%)	0	0
				TUPLE1-SHGC-2421	Atypical deletion	Normal	1 (1.3%)	0	0	0	0	1 (1.3%)
						Abnormal	0	1 (1.3%)	1 (1.3%)	1 (1.3%)	1 (1.3%)	0
				TUPLE1-D22S936	Atypical deletion	Normal	1 (1.3%)	1 (1.3%)	1 (1.3%)	1 (1.3%)	1 (1.3%)	1 (1.3%)
						Abnormal	0	0	0	0	0	0
				ZNF74-D22S936	Atypical deletion	Normal	0	1 (1.3%)	1 (1.3%)	1 (1.3%)	1 (1.3%)	1 (1.3%)
						Abnormal	1 (1.3%)	0	0	0	0	0
**TD**	15	M = 8 F = 7	M = 8-14 F = 9-13	N/A	N/A	N/A	N/A	N/A	N/A	N/A	N/A	N/A

Demographic information, size of the deletion, Number and percent of individuals presenting with specific clinical involvement including IQ, ADHD, seizures, CHD, hypocalcemia and thyroid abnormalities are shown.

### Clinical measures

All participants with 22q11DS were examined by a developmental behavioral pediatrician (DBP) or child and adolescent psychiatrist and also underwent neuropsychological assessment, including FSIQ using the WISC-4 [46]. FSIQ ≤70 (2 standard deviations below normed means) was used to define abnormally low FSIQ in line with accepted classifications for mental retardation/intellectual disability. Presence of physical conditions (neurologic, cardiac, endocrine, etc.) was obtained from medical history from the parents during the visit with the DBP or psychiatrist, including current and past medications, and when available, medical record abstraction. Examples of abnormal medical conditions included seizures and congenital heart disease such as Tetralogy of Fallot, Truncus Arteriousus, and septal defects. Endocrine disturbances such as hypocalcemia and hyper/hypothyroidism were also documented. Participants were considered to have ADHD if scores on the parent-completed SNAP-IV [47] were above the 95% cutoff for ADHD-inattentive, ADHD-hyperactive, or ADHD-combined type. All TD participants had FSIQ > 85 on the WASI [48].

### DNA isolation

Genomic DNA (gDNA) from 80 individuals with 22q11DS and 15 TD individuals was isolated from 3–5 mL of peripheral blood leukocytes using standard procedure (Qiagen, Valencia, CA).

### ddPCR

ddPCR involves partitioning the PCR reaction mix into uniform-size droplets, thermal cycling to end-point fluorescence and then singulating and reading the fluorescence of each droplet. Using an assay specific to amplify the DNA targets of interest will result in a fluorescent signal derived from the droplets that contain the DNA target while no signal will be detected from those that do not contain target DNA. Thus, each droplet is counted and able to report an actual number of copies in the sample. For the TaqMan reactions, 2 μg of gDNA was digested at 37°C for 1 hour, with MseI in NEB Buffer 2.1, in a final volume of 10 μL. An assay mix containing 100 ng of digested gDNA, Droplet PCR supermix (BioRad Laboratories, Hercules, CA), and gene assay at a final concentration of 900 nM per primer and 250 nM probe in a 25 μL final volume was prepared. For the EvaGreen reactions, an assay mix containing 100 ng of gDNA, 5 units MseI, QX200 ddPCR EvaGreen supermix (BioRad Laboratories, Hercules, CA), and gene assay at a final concentration of 100 nM per primer in a 25 μL final volume was prepared. 20 μL of assay mix and 70 μL of ddPCR droplet oil (BioRad Laboratories, Hercules, CA) were transferred onto a QX100/200 DG cartridge (BioRad Laboratories, Hercules, CA), then loaded into the QX100 Droplet Generator (BioRad Laboratories, Hercules, CA). Vacuum was applied, pulling individual samples and oil through a flow-focusing junction to produce ~20,000 water-in-oil droplets. 40 μL of the oil and sample droplet emulsions were then transferred into a 96 well plate and thermocycled in a standard themocycler (BioRad Laboratories, Hercules, CA) for 95°C for 10 minutes, 94°C for 1 min and 59°C for 1 min (repeated 40 times) and 98°C for 10 minutes (TaqMan reactions) or 95°C for 5 minutes, 96°C for 30 seconds and 60°C for 1 minute (repeated 40 times), 4°C for 5 minutes and 90°C for 5 minutes (EvaGreen reactions). The plate was then transferred to a QX200 Droplet Reader (BioRad Laboratories, Hercules, CA) and analyzed by QuantaSoft (BioRad Laboratories, Hercules, CA). For the TaqMan assays, the presence of 2 copies of any given gene region was scored by a CNV value of 2 ± 0.1 and a hemizygous deletion (1 copy) of a region was scored by a CNV value of 1 ± 0.1. For the EvaGreen assays, a hemizygous deletion of a region was scored by a CNV value of 1 ± 0.4. TaqMan target primers were designed as described by Weksberg et al. [35]. *D22S181, PRODH*, and *D22S936* were specifically chosen to help elucidate the position of the deletion breakpoints. *RPP30*, a reference assay commonly used in ddPCR CNV studies, as it is located in a conserved region on the genome not known to undergo copy number variation, was used as the control [44]. The control probe (*RPP30*) was labeled with VIC and all target probes were labeled with FAM unless otherwise described. TaqMan and EvaGreen primer sequences were as shown in Tables 2 and 3.

**Table 2:** **TaqMan primer sequences**

**Gene**	**Forward primer**	**Reverse primer**
D22S182	5′- CAGCTCCCAAGTCTTTCCAGC -3′	5′- CCAGGGTAGGAAACAGGTCGA -3′
PRODH	5′- GGGAAAGGAGAGTTCAGGCAG -3′	5′- GCTTGTTGAATAGCCTCTGTCCTAG -3′
TUPLE1	5′- GGCAAGTGCAATATTCATGTGGT -3′	5′- TCCTACACGCCTGACAAAGCT -3′
COMT	5′- GTGCTACTGGCTGACAACGTGAT -3′	5′- GGAACGATTGGTAGTGTGTGCA -3′
ZNF74	5′- TGGCCTCCTGCTTCTTTCTTC -3′	5′- CAGACACTCCAATTCATGACGAA -3′
PIK4C4	5′- ATGCTTGTGCGACGCAGAC -3′	5′- CCTCAGCCATGTTGACTCAGC -3′
D22S936	5′- TGGCAGCCAGTTTAGTATTCTGC -3′	5′- TTGTAATCAAGTCCCGCCACT -3′
VPREB1	5′- CGACCATGACATCGGTGTGT -3′	5′- CTGGCTCTTGTCTGATTGTGAGA -3′

**Table 3:** **EvaGreen primer sequences**

**Gene**	**Forward primer**	**Reverse primer**
DGCR8	5′- ATGTGTTCCTTCTGCTCTGAT- 3′	5′- CTTACTACAGAGGAAGCATGAAG - 3′
TRMT2A	5′- TTTTGCTCACCCTTCCTGTT- 3′	5′- CTGGCAGTCAAACAAGAGGA- 3′
RANBP1	5′- GAGTGCAGCAGTGGTATCAT- 3′	5′- GATGGCTAACACCCGTAGTC- 3′
ZDHHC8	5′- CTTTCATGGACCCTGGTGTT- 3′	5′- TCCCTAAGGCTGTCTCAAGT- 3′
LOC284865	5′- GCCTTGACCTCTGTTTCTGT- 3′	5′- CCCAAGAAGAAAGAGGCACA- 3′
RTN4R	5′- TGATGTGAGAAGGTCCTCCA- 3′	5′- CTGCTTCCCTCAGTTGGAAA- 3′
DGCR6L	5′- AGTGTTCGGAAGAGGTCTCT- 3′	5′- AACAAAACTGGTTGGACCCA- 3′
SCARF2	5′- TAGGGCCAGTCTATCCCATC- 3′	5′- TTCACAAGCAGGCTTGGATT- 3′
SHGC-2421	5′- TCATGTGGGTGCTGGTAC- 3′	5′- TCCTTGCACCAGGCAAC - 3′
HIC2	5′- GAGTCCCTCAGAGAATGGC- 3′	5′- GCCCTGTGGAAGCCTG- 3′

**Figure 1:**
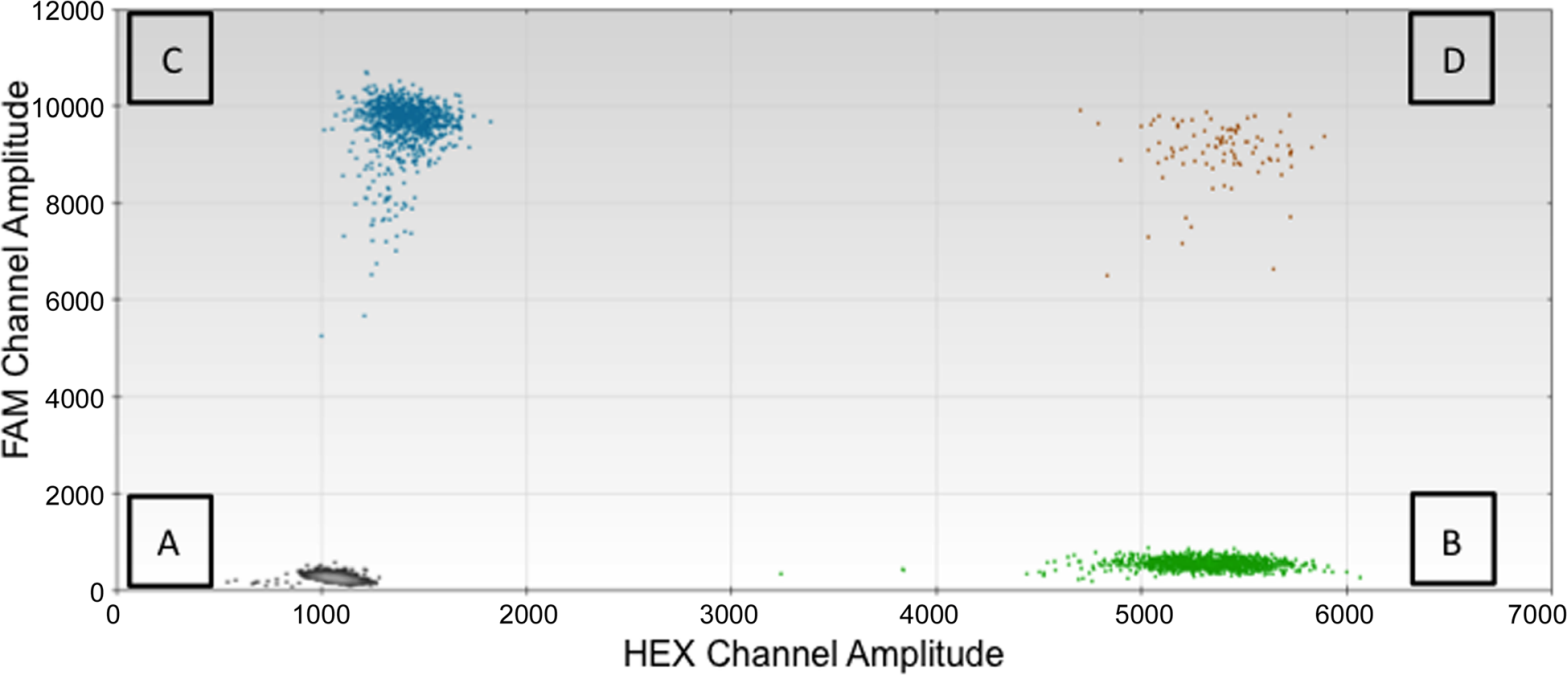
**2D Fluorescent Amplitude Plot.** Cluster **(A)** comprises the double negative droplets (droplets containing no amplicons). Cluster **(B)** contains droplets containing the reference amplicon. Cluster **(C)** contains droplets containing the target amplicon. Cluster **(D)** comprises the double positive droplets (droplets containing both target and reference amplicons).

To calculate CN measurements for the TaqMan assays, automatic thresholding in Quantasoft was performed on the 2D fluorescent amplitude plot (Figure 1). CN was calculated as (a/b)*c where “a” is the copy of DNA target gene (assays 1–8) per microliter, “b” is the copy of the reference gene (RPP30) per microliter and “c” is the CN of the reference gene per genome. Average CN values for all assays are reported in Table 4. To calculate CN measurements for the Evagreen assays, CN was calculated as (d/e)*2 where “d” is the concentration of the target assay and “e” is the concentration of the reference assay (Table 5).

**Table 4:** **Copy number values for TaqMan-based ddPCR assays**

**Assay**	**TD**		22q11DS*	
	**Average**	**Std Err**	**Average**	**Std Err**
D22S181	2.04	0.03	2.02	0.05
PRODH	2.09	0.08	1.03	0.12
TUPLE1	2.03	0.04	1.02	0.12
COMT	2.06	0.07	1.02	0.12
ZNF74	1.96	0.04	0.97	0.16
PIK4CA	2.03	0.03	1.03	0.12
D22S936	2.05	0.07	1.02	0.12
VPREB1	2.04	0.08	2.08	0.44
**Average**	**2.04**	**0.09**	**1.02**	**0.13**

*22q11DS values are only shown for individuals with the 3 Mb deletion spanning the region from *PRODH*-*D22S936.*

**Table 5:** **Copy number values for EvaGreen-based ddPCR assays**

**Assay**	**Average**	**Std Err**
DGCR8	1.02	0.32
TRMT2A	1.07	0.47
RANBP1	1.06	0.39
ZDHHC8	1.00	0.37
LOC284865	1.02	0.34
RTN4R	1.02	0.37
DGCR6L	1.04	0.36
SCARF2	1.24	0.53
SHGC-2421	1.37	0.44
HIC2	1.83	0.12
**Average**	**1.09**	**0.40**

### Quantitative PCR

Genomic DNA (gDNA) in a subgroup of 40 participants with 22q11DS was analyzed by qPCR using the methods described in Weksberg et al. [35] with minor modifications. Target assays used from Weksberg et al. [35] were the same as those used for ddPCR and reference assays used were *HEM3* and *G6PDH* (2005). For each sample, quantitative-PCR reactions were performed in two independent runs in duplicates. Control reactions were run in parallel. Reactions were performed using FastStart Universal SYBR Green Master Mix (Roche, Applied Science, Indianapolis, IN) (which includes the internal reference (ROX), forward and reverse primers at final concentrations of 800 nM for the target primers and 400 nM for the reference primers, and 10 ng of genomic DNA. The qPCR reactions were run using the Applied Biosystems 7900HT FAST real-time PCR system (Foster City, CA) with 2 min at 50°C, 10 min at 95°C followed by 40 cycles of 15 sec at 95°C and 60 sec at 60°C.

### Statistical analysis

QuantaSoft software (version 1.3.2) (Bio-Rad Laboratories, Hercules, CA) was used to analyze the ddPCR data and to calculate the copy number estimations. qPCR data was analyzed using the comparative Ct method after data normalization [35].

## Results and discussion

### Deletion endpoints

**Figure 2:**
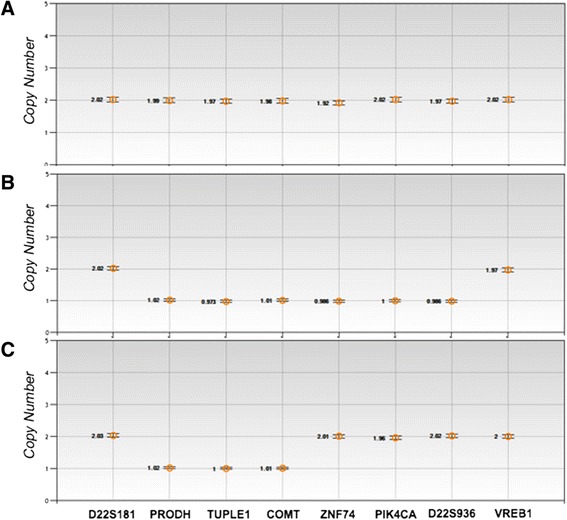
**Diagram of CNV values by QuantaSoft.** CNV values for **A)** a TD individual **B)** an individual with a 3 Mb deletion and **C)** an individual with a nested 1.5 Mb deletion are represented by each of the 8 TaqMan assays noted at the bottom. 95% Confidence Interval are shown for each sample.

**Figure 3:**
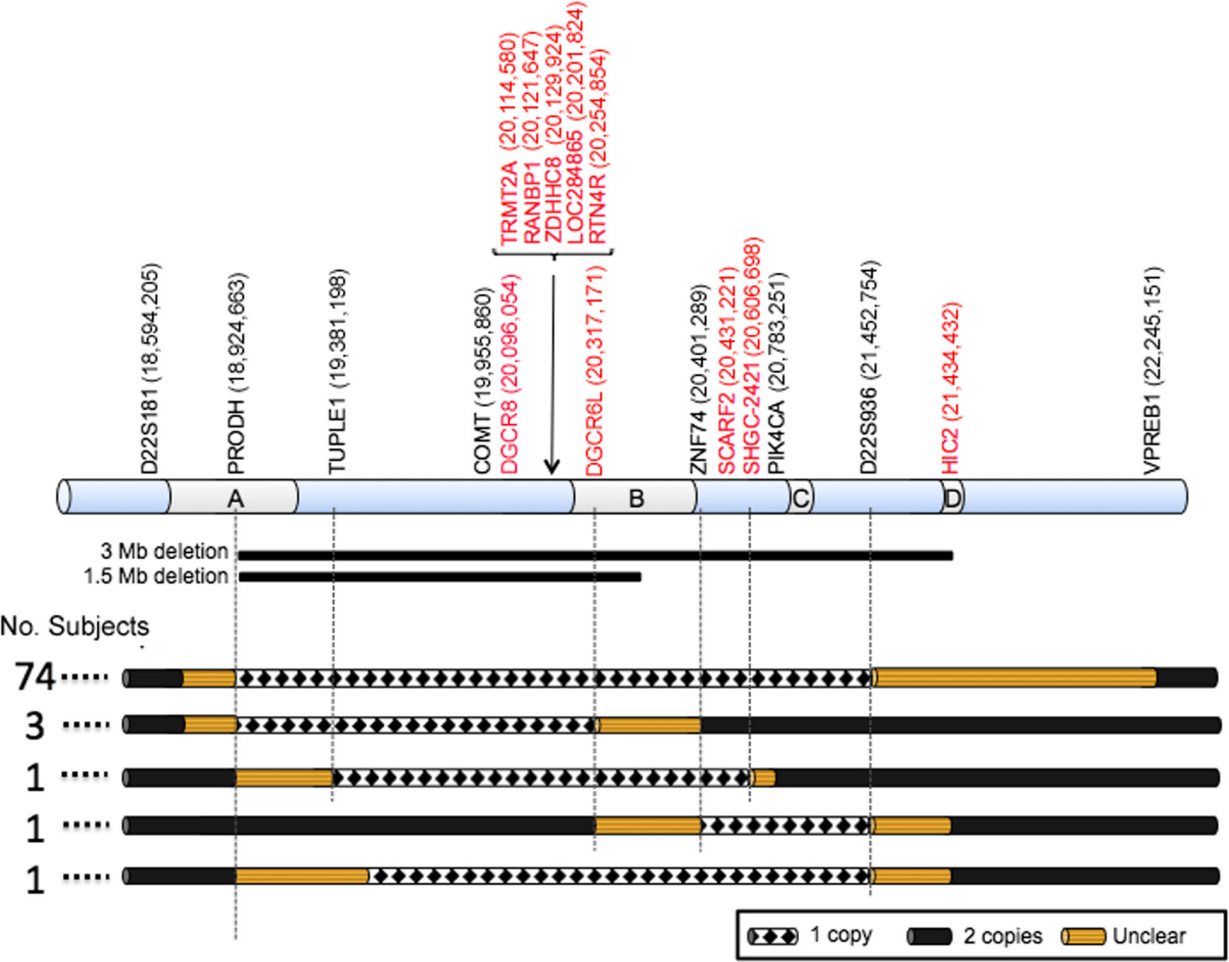
**Sizes and types of chromosome 22 deletions.** The diagram summarizes the deletions identified in the 80 participants of the study. Diamond regions indicate a hemizygous deletion of one gene copy; solid dark regions indicate the presence of two copies of chromosome 22; solid light regions indicate uncertain areas of deletion. Locations of assays in base pairs are noted (as reported in the UCSC Genome Browser, 2013). Numbers of individuals are noted on the left. Genes in black indicate TaqMan assays and genes in red indicate EvaGreen assays.

To characterize the size and the location of deletions of 80 individuals known to have 22q11DS by FISH, we used ddPCR to define their deletion endpoints. In this study, 15 typically developing (TD) individuals were used as controls in this study. Our analysis involved 8 loci spanning an approximately 4 Mb region of chromosome 22q11.2. The two outermost genes are thought to be unaltered in individuals with 22q11DS (*D22S181* and *VPREB1*). In all cases, 2 copies of chromosome 22 were detected in the TD (Figure 2A) and either the 3 Mb (Figure 2B) or 1.5 Mb (Figure 2C) deletions were identified in the individuals with 22q11DS with no ambiguity. We found that 74 individuals carried the 3 Mb deletion and had 1 copy of the 6 genes located within the deleted region and 2 copies of the 2 genes located outside (*D22S181* and *VPREB1*; Figure 2B,C and Figure 3). The remaining 6 individuals with 22q11DS, 3 with the 1.5 Mb deletion and 3 with an interstitial (non-contiguous interspersed deletions) deletion presented with 1 copy of those genes located within the specific deleted region (Figure 2C, and Figure 3). Finally, 15 typically developing (TD) control individuals showed the presence of 2 copies of the 8 genes tested except for one individual who showed a duplication of the *PRODH* gene. The types of deletion found in the individuals in our study samples represent the expected distribution in the 22q11DS population, as we found 92% of individuals having the 3 Mb deletion, 4% having the 1.5 Mb deletion, and 4% having atypical deletions. The majority of individuals had deletions encompassing PRODH and D22S936 with deletion breakpoints near or within LCR-A and LCR-D. None of the 80 samples had a distal deletion extending beyond the LCR-A and LCR-D region, supporting the hypothesis that the majority of 22q11DS individuals have deletion breakpoints located near or within those regions.

Interestingly, three individuals carried interstitial deletions of different sizes and locations, likely originating from more rare events involving smaller LCRs present throughout the 22q deleted region. To perform higher resolution mapping and to narrow down the exact deletion breakpoints, we used ten additional EvaGreen assays to clarify the deletion region for the six individuals with the less common deletions (Figure 3). Specifically, the three individuals with the 1.5 Mb deletion showed a deletion between *PRODH* and *DGCR6L*, and the three individuals with the atypical deletions showed a deletion between *TUPLE1* and *SHGC-2421, ZNF74* and *D22S936*, or *TUPLE1* and *D22S936* (Figure 3). Average copy number values are reported in Tables 4 and 5.

### Comparison between qPCR and ddPCR

**Figure 4:**
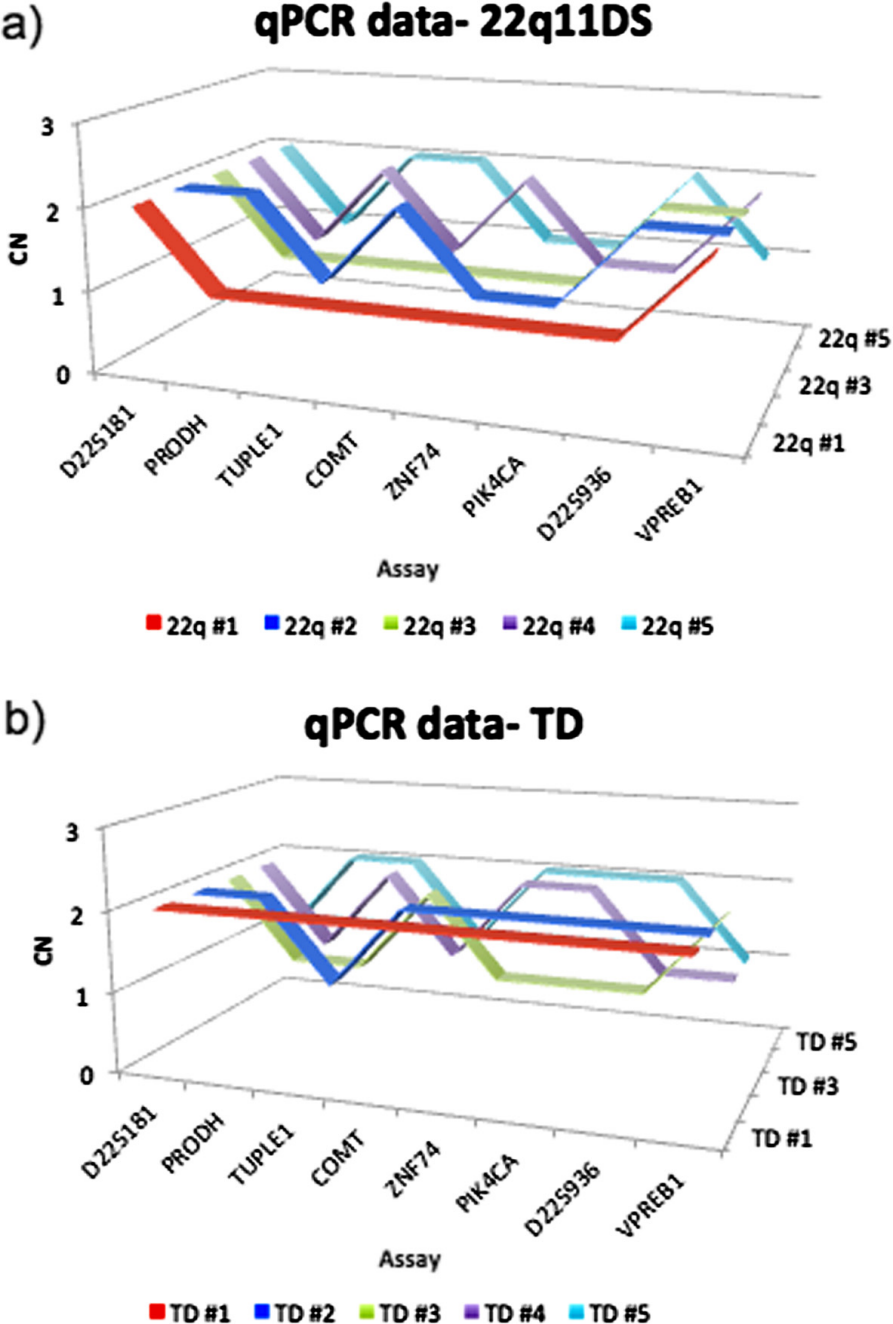
**qPCR (SYBR Green) vs. ddPCR (TaqMan) results.** Graphic representation of qPCR results for five representative cases for **A)** individuals with 22q11DS and **B)** TD individuals across all 8 assays used in ddPCR (TaqMan). The first individual for each graph represents one where qPCR and ddPCR results were concordant. The y-axis indicates the copy number and the x-axis indicates the position of the 8 assays used in the analysis.

To determine whether ddPCR offers advantages, particularly in performance, reliability and specificity, over qPCR for 22q11DS detection, we analyzed a subgroup of 40 individuals with 22q11DS by qPCR (SYBR Green) using the same eight assays used for ddPCR (Taqman) analysis. qPCR data showed that 18 (53%) of these individuals had non-contiguous interstitial deletions, which were determined to be contiguous by ddPCR. Of the individuals tested, 2 (6%) were determined to have a larger deletion than that resolved by ddPCR and 4 (12%) were determined to have smaller deletions than that resolved by ddPCR. Only 6 (15%) of the qPCR deletion endpoint determinations were the same as those obtained with ddPCR (Figure 4). In respect to the copy number in the *TUPLE1* gene (for comparison with FISH data), ddPCR results for 80 individuals with 22q11DS and 15 TD individuals was found to be 100% specific (Table 6), whereas qPCR results for 19 individuals with 22q11DS and 11 TD individuals was only 58.6% specific (Table 7). Thus, in our hands, qPCR failed to accurately define the deletion breakpoints, possibly due to its reliance on standard curves and its sensitivity to amplification efficiencies and thus, it may not provide sufficient accuracy to serve as a comprehensive diagnostic tool.

**Table 6:** **Specificity of diagnosis calls by TaqMan-based ddPCR**

**Outcome ddPCR assay**	**Positive**	**Negative**	**Total**
**Positive**	80	0	80
**Negative**	0	15	15
**Total**	80	15	95

Results obtained by TaqMan-based ddPCR are compared to the diagnosis of 22q11DS obtained by FISH using the TUPLE1 gene Specificity was 100% as all 80 individuals were correctly identified as having 1 copy of the TUPLE1 gene.

**Table 7:** **Specificity of diagnosis calls by qPCR**

**Outcome qPCR assay**	**Positive**	**Negative**	**Total**
**Positive**	17	0	17
**Negative**	12	11	23
**Total**	29	11	40

Results obtained by qPCR are compared to the diagnosis of 22q11DS obtained by FISH using the TUPLE1 gene. Specificity of diagnosis calls for qPCR was 58.6% as ddPCR results, conducted in a subgroup of 40 individuals (29 with 22q11DS and 11 TD) correctly identified 1 copy of the *TUPLE1* gene only in 17 individuals with 22q11DS.

Advantages of TaqMan-based ddPCR include no need for a standard curve, multiple targets and reference gene are measured in the same well and greater tolerance to differences in amplification efficiencies, which together result in much more robust CN measurements. Furthermore, our data indicates the assays used in this study have a specificity of 100% (95% Confidence Interval = 95.5-100%), allowing us to delineate deletion sizes and locations with great certainty, making it a preferred tool for diagnostics.

### Genotype-phenotype correlations

The ddPCR data on the deletion endpoints was used to investigate whether the deletion type/size correlated with various clinical phenotypes including IQ, ADHD, seizures, CHD, hypocalcemia, and thyroid abnormalities (as listed in Table 1) to potentially narrow down a minimal region for disease in the subjects included in this study. The majority of individuals in our 22q11DS cohort (~62%) had higher (>70) than average IQ scores with a mean score of 73 (±13), while ADHD was diagnosed in 38% of them. Thyroid abnormalities, seizures and CHD were seen in 14%, 21% and 38% of the participants, respectively. Examination of the clinical phenotypes present in all individuals indicated that many of the clinical features were present in those with the 1.5 Mb deletion defined between locus *PRODH* and *ZNF74* (LCR-A and LCR-B). Interestingly, one of the individuals with an atypical deletion mapping approximately between locus *ZNF74* and *D22S936* (LCR-B and LCR-D), had ID while another individual with an atypical deletion mapping approximately between locus *TUPLE1* and *SHGC-2421* presented with ADHD, seizures, CHD and hypocalcemia and another individual with an atypical deletion mapping between locus *PRODH* and *DGCR6L* presented with seizures and CHD. In addition, the three individuals with the nested deletion (1.5 Mb) had ID and ADHD and one had a more severe phenotype including CHD and seizures. This indicates that other factors (in addition to the 22q11 deletion) are playing a role in the broad variation of the observed phenotype. However, this region of chromosome 22 contains many genes including *HIRA*, *TBX1*, and *DGCR8*, all of which are thought to be candidate genes for CHD. Although *TBX1* is thought to be the key player for CHD, the presence of individuals with 22q11DS with CHD but without a *TBX1* deletion and the lack of pathogenic mutations identified in the gene suggest that deletion of *TBX1* alone, is not sufficient to give rise to CHD [17,49-51]. Our findings confirm previous reports [32,39] indicating that the correlation between the size or/and position of the deleted region and the broad spectrum of phenotypes is not straightforward and that other factors play a role in contributing to the phenotypes observed in 22q11DS.

One of the genes mapping to the deleted region of chromosome 22q11.2 is *DGCR8,* a gene expected to affect multiple genes as it plays an important role in microRNA (miRNA) biogenesis. miRNAs are small non-coding RNA molecules that are initially transcribed by RNA Polymerase II as primary miRNAs transcripts; then processed into precursor miRNAs by DROSHA and DGCR8 (which anchors DROSHA to the primary miRNA) [52-56]. Precursor miRNAs are then exported into the cytoplasm where they are processed into mature miRNAs by the DICER enzyme [57-59]. miRNAs are post-transcriptional gene expression regulators, and can inhibit mRNA translation or promote mRNA degradation [57]. Therefore, hemizygous deletion of *DGCR8* could alter the expression of multiple genes. Several studies in mice and humans have demonstrated a possible correlation of miRNA dysregulation with CHD phenotype in 22q11DS [60-62].

Finally, building genotype-phenotype correlations in 22q11DS can allow for better interventions in individuals with 22q11DS. Similarly, they also function to provide an understanding of the role of the genes in these deleted regions in disorders like ADHD and schizophrenia.

## Conclusions

In this study we demonstrate the feasibility of ddPCR for detecting and mapping deletions of chromosome 22q11.2. While other methods have been used to elucidate deletion endpoints in individuals with 22q11DS, none have all the qualities required for an effective and reliable diagnostic tool. Additionally, clinical and molecular diagnostics require methodologies that are robust, cost effective and at high throughput.

Although it is not common medical practice to identify the extent of patients’ deletions or the location, it may be important, in some cases, to better characterize the LCRs and individual’s deletion, size and location. However, many of the genes involved in clinical phenotypes in 22q11DS appear to be located in the common 1.5 Mb deletion region between LCR A-B.

Our findings support the hypothesis that the majority of individuals with 22q11DS have deletion breakpoints located near or within LCRs A and D. Two individuals appear to have breakpoints localized within smaller LCRs scattered along chromosome 22.

Importantly, in this study we show that ddPCR is an ideal technology for detecting 22q11DS and can allow fine mapping of the deleted region using either TaqMan or EvaGreen assays and may be qualified for use in a clinical setting. ddPCR is a high throughput, cost effective technology that allows absolute measure of nucleic acid concentration, providing highly accurate estimations of DNA copy number. Approximately 200 samples can be easily run and analyzed daily, without the difficulties, ambiguity, time and cost of other methodologies, making ddPCR the preferred tool for determining DNA copy number variation in large population screening studies.

## Abbreviations

22q11DS: 22q11.2 Deletion Syndrome
ddPCR: droplet digital PCR
CN: Copy Number
CHD: Congenital Heart Disease
ID: Intellectual Disabilities
ADHD: Attention Deficit Hyperactivity Disorder
LCR: Low Copy Repeats
FISH: Fluorescent *In-Situ* Hybridization
MLPA: Multiplex Ligation-Dependent Probe Amplification
qPCR: quantitative PCR
aCHG: array comparative genomic hybridization
TD: Typically Developing Controls
miRNA: microRNA
